# Minimally invasive laser enucleation of the prostate (MiLEP) in a patient with prior EPA urethroplasty and an indwelling “Butterfly” prostatic retractor device: A case report

**DOI:** 10.1016/j.eucr.2026.103440

**Published:** 2026-04-09

**Authors:** Ophir Winder, Itay Sabler, Pavel Bakaleyschik, Amir Zarror, Amnon Zisman, Ehud Gnessin

**Affiliations:** a Assuta Medical Center, 20 HaBarzel St., Ramat HaHayal, Tel Aviv, 69710, Israel; b Department of Urology, Shamir Medical Center, Zerifin, 70300, Israel

**Keywords:** Benign prostatic hyperplasia (BPH), Holmium laser enucleation of the prostate (HoLEP), Minimally invasive HoLEP (MiLEP), Butterfly prostatic device, Urethral stricture, Nitinol implant extraction

## Abstract

We present the first reported case of holmium laser enucleation of the prostate (HoLEP) in a patient with a mucosa-embedded nitinol-based “Butterfly” prostatic retractor device and a prior Excision and Primary Anastomosis urethroplasty for bulbar stricture. Using a 22Fr minimally invasive resectoscope (MiLEP), the device was fragmented with the laser and extracted in its entirety. Prostatic enucleation and morcellation were successfully and safely completed. Morcellation is feasible but requires caution due to device interference. This study demonstrates the feasibility of small-caliber MiLEP in patients with complex anatomy and implanted prostatic devices, expanding minimally invasive options for surgically challenging BPH cases.

## Highlights

• First HoLEP performed with an in-situ “Butterfly” prostatic device.
• 22Fr MiLEP safely performed after prior EPA urethroplasty.
• Laser fragmentation allowed safe extraction of the embedded nitinol device.
• Morcellation is feasible but requires caution due to device interference.
• MiLEP is feasible in patients with complex anatomy and implanted prostatic devices.

## 1 Introduction

Benign prostatic hyperplasia (BPH) is a common and progressive condition among aging men, often resulting in benign prostatic obstruction (BPO) and bothersome lower urinary tract symptoms (LUTS) that can significantly impact quality of life.^1^

Over the last decade, several minimally invasive surgical therapies (MIST) have emerged to bridge the gap between pharmacotherapy and more invasive procedures 1, 2, 3, aiming to relieve symptoms and improve urine flow while reducing morbidity and preserving sexual function. They also provide a valuable alternative for patients who are either unfit for or unwilling to undergo conventional surgery, as well as those seeking treatments that minimize the risk of ejaculatory or erectile dysfunction.^1^

The Butterfly Prostatic Retraction device (“Butterfly” Medical, Yokneam, Israel) is a novel, minimally invasive, reversible, non-surgical treatment designed to dilate the prostatic urethra and treat LUTS.^2^

**Fig. 1:**
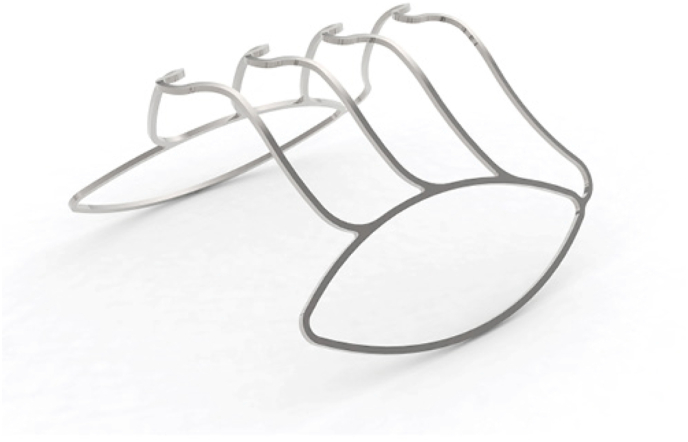
**“Butterfly” Prostatic Retractor Device.** The “Butterfly” is a permanent nitinol implant shaped like a butterfly, consisting of two lateral wings connected by transverse arches.

The “Butterfly” is a permanent nitinol implant shaped like a butterfly, consisting of two lateral wings connected by transverse arches (Fig. 1). This device is deployed transurethrally, under local anesthesia. Upon deployment, its spring mechanism expands to retract the lateral prostatic lobes. In a pilot study by Katz et al., “Butterfly” treatment significantly improved LUTS and urinary flow at 12 months, with no reported erectile or ejaculatory dysfunction. Postoperative cystoscopy demonstrated gradual coverage of the device by urethral mucosa. Consequently, in patients experiencing dissatisfaction or discomfort (approximately 30% of the cohort), early device removal within the first 6 months is possible before mucosal overgrowth.^2^ Katz et al. described device extraction via rigid cystoscopy using chilled saline (5–10 °C) to soften the nitinol structure, exploiting its thermal properties. The anterior arch of the device was grasped with alligator forceps and withdrawn through the rigid cystoscope sheath.^2^

Holmium laser enucleation of the prostate (HoLEP) is a well-established treatment for BPH, demonstrating superior efficacy and durability compared to other transurethral interventions. According to the recent American Urological Association (AUA) guidelines on the surgical management of BPH/LUTS, HoLEP should be offered as a prostate size–independent treatment option for patients with BPH.^3^^,^^4^ Traditionally, HoLEP is performed using a 26-28Fr resectoscope. Recently, Minimally Invasive HoLEP (MiLEP) using 22Fr resectoscopes has been introduced. Smaller caliber resectoscopes are hypothesized to reduce urethral trauma. However, this transition has raised concerns regarding irrigation pressure, outflow efficiency, and visualization quality. Nevertheless, recent retrospective studies have demonstrated that MiLEP is a safe and feasible procedure.^5^^,^^6^

Herein, we report a successful 22Fr MiLEP in a 69-year-old patient with a mucosa-embedded “Butterfly” device and a concomitant grade 1 bulbar urethral stricture following excision and primary anastomosis (EPA) urethroplasty.

## 2 Case presentation

A 69-year-old male presented with symptomatic BPH. He underwent insertion of a “Butterfly” device in 2020, after which he developed a urethral stricture that was successfully treated with EPA urethroplasty. Despite undergoing both procedures, symptoms worsened, and the lower urinary tract issues remained unresolved.

Cystoscopy performed one year after urethroplasty demonstrated a patent urethral anastomosis and an obstructing, irregular prostate with a prominent median lobe protruding into the bladder.

In 2025, the patient presented severe voiding and storage symptoms, significantly impairing quality of life (IPSS 25, bother index 6). His primary complaints were worsening obstructive symptoms and increased post-micturition residual volume (PVR). In addition to a significant impairment in sexual function since the surgical interventions. His symptoms were refractory to combination medical therapy, including alfuzosin, tamsulosin, and tadalafil. Ultrasound demonstrated a 57-cc prostate volume and a 200-cc PVR. Uroflowmetry depicted a low maximum flow rate (Qmax) of 5 cc/s, a 270-cc voided volume, and a 134-cc PVR. The patient was otherwise healthy, with no significant medical history. Physical examination and PSA levels were within normal limits.

Preoperative flexible cystoscopy demonstrated a normal penile urethra. A grade 1 circumferential bulbar urethral stricture with an adjacent false passage partially obstructing the lumen was noted. The urethral anastomosis was patent. An additional false passage was observed anterior to the bladder neck, although entry into the bladder was unimpeded. The prostate appeared irregular and obstructive despite the presence of the "Butterfly" device, with a prominent median lobe. The bladder was trabeculated, with normal-appearing ureteral orifices.

In summary, the patient had a minor grade 1 bulbar urethral stricture following EPA urethroplasty and presented with symptomatic obstruction at the prostate level in the presence of a "Butterfly" device. We offered relief of the prostatic obstruction via HoLEP, combined with the extraction of the "Butterfly" device. The patient was counseled regarding the risk of secondary urethral stricture, particularly if a 24–26 Fr resectoscope would be required for device removal. In a shared decision-making process, we planned to perform HoLEP using a 22 Fr resectoscope (MiLEP) with an attempt to extract the Butterfly device through the same instrument. If extraction could not be achieved, conversion to a 24–26 Fr instrument or to an open procedure was planned.

**Fig. 2:**
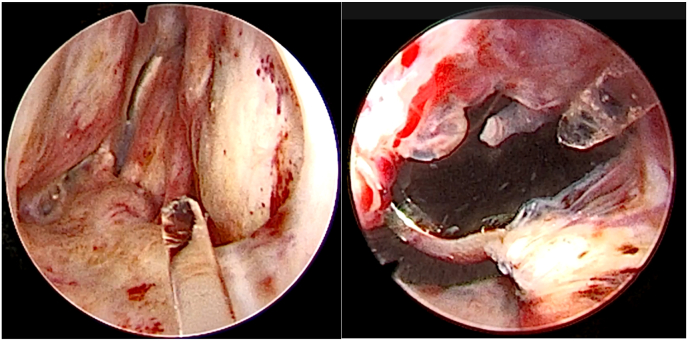
**Exposure of the “Butterfly device”.** Left- Cystoscopic appearance of prostate at the beginning of surgery. Right- The exposed U-shaped posterior part of the “Butterfly”.

**Fig. 3:**
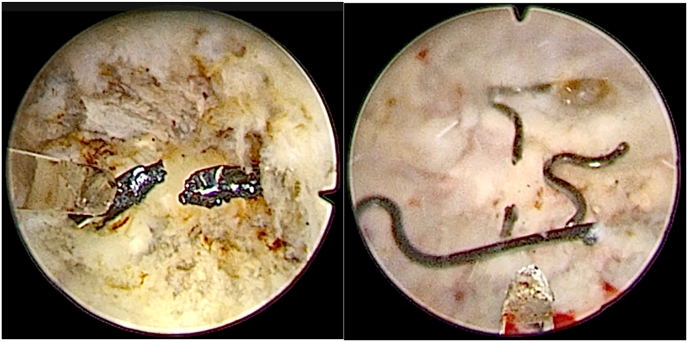
**‘Butterfly’ device following laser fragmentation.** Left: In-situ ‘Butterfly’ device following laser fragmentation. Right: Fragments of the ‘Butterfly’ device following laser treatment.

The patient underwent the planned surgery successfully: 22Fr (MiLEP) with extraction of the “Butterfly” device. Prophylactic intravenous antibiotics were given (Cefazolin and Gentamicin). Surgery commenced with a 19Fr cystoscopy, identifying the grade 1 bulbar urethral stricture at the urethral anastomosis, which did not require dilatation. A hydrophilic guidewire was passed through the stricture, and a 22Fr urethral dilator was used. The urethral lumen was obstructed at the prostatic level, and the "Butterfly" device was not seen as it was embedded deep within the prostate tissue (Fig. 2, Video 1). A 22 Fr Holmium:YAG laser resectoscope (“Moses 2.0”, 550 μm fiber, energy up to 100 W) was then used. The “white line” was marked by incising the urethral mucosa immediately anterior to the sphincter to release the sphincter mucosa from the prostate. Incisions were made anteriorly and laterally to the verumontanum, and at 12 o'clock in the prostatic urethra. En-bloc enucleation of the prostate tissue was completed, and the adenoma together with the butterfly device were pushed into the bladder. During enucleation, the “Butterfly” device was exposed and easily incised with the laser (Fig. 2, Fig. 3). Enucleation required approximately 50 minutes, followed by 10 minutes of morcellation using a Piranha morcellator.

Supplementary data related to this article can be found online at https://doi.org/10.1016/j.eucr.2026.103440

The following are the Supplementary data related to this article:

Video 1Exposure of mucosa-embedded “Butterfly” device via MiLEP .Multimedia component 1

During morcellation, one morcellator blade tooth fractured against the nitinol implant and was retrieved with alligator forceps. A new morcellator blade was then used cautiously. A fragment of the nitinol device became lodged in the morcellator and was removed manually. The “Butterfly” is made of temperature-responsive nitinol; thus, cooling it with saline kept it flexible and allowed easier removal of device fragments using a 22Fr resectoscope and alligator forceps. Meticulous hemostasis was achieved, and a second cystoscopic inspection confirmed complete removal of all device fragments and prostatic tissue.

A 3-way 20 Fr Foley catheter, with 50 mL saline in the balloon, was inserted. Continuous bladder irrigation was initiated, producing clear urine output. The procedure was completed uneventfully, with no significant blood loss or intraoperative complications. The patient experienced an uneventful postoperative course. On postoperative day 1, the catheter was removed upon clear urine output, and the patient was discharged home following successful voiding.

## 3 Discussion

To the best of our knowledge, this is the first HoLEP reported on a patient with the novel “Butterfly” device in situ. The case was particularly challenging as we used a small 22Fr resectoscope (MiLEP) to minimize the risk of urethral injury, given the patient's bulbar urethral stenosis following EPA urethroplasty.

This case is unique for several reasons. First, although the “Butterfly” device manual recommends extraction with a 26–28 Fr sheath, we demonstrated that safe extraction was achieved using a 22 Fr resectoscope after laser fragmentation, despite a grade 1 bulbar stricture. Second, we showed the feasibility of performing MiLEP with a small 22Fr resectoscope even in a patient with grade 1 bulbar urethral stenosis following EPA urethroplasty. Third, we demonstrated that prostate laser enucleation is possible in the presence of the nitinol “Butterfly” device, using the Ho:YAG “Moses™ 2.0” laser, which employs unique pulse modulation and requires a specially designed fiber.^3^ Fourth, we confirmed that morcellation is achievable but less efficient in the presence of the nitinol device. This case required changing the morcellator blade. Morcellation should therefore be approached with caution in patients carrying this device.

## 4 Conclusion

This case demonstrates that MiLEP via a 22 Fr resectoscope is feasible in patients carrying a “Butterfly” device, even following EPA urethroplasty for urethral stricture. This device can be safely removed after laser fragmentation, and prostate enucleation can be performed using the Ho:YAG laser with the “Moses™ 2.0” system. Morcellation, though less efficient, is possible with careful technique. This report highlights the safety, adaptability, and utility of small-caliber MiLEP in complex anatomical and device-related scenarios.

## CRediT authorship contribution statement

**Ophir Winder:** Conceptualization, Formal analysis, Investigation, Methodology, Project administration, Writing – original draft, Writing – review & editing. **Itay Sabler:** Formal analysis, Investigation, Project administration, Writing – review & editing. **Pavel Bakaleyschik:** Investigation. **Amir Zarror:** Investigation. **Amnon Zisman:** Formal analysis, Project administration, Validation, Writing – review & editing. **Ehud Gnessin:** Conceptualization, Data curation, Methodology, Project administration, Supervision, Writing – review & editing.

## Disclosures

This research did not receive any specific grant from funding agencies in the public, commercial, or not-for-profit sectors.

Informed consent was obtained.
